# American Dental Society of Europe

**Published:** 1884-11

**Authors:** W. St. Geo. Elliott

**Affiliations:** London


### AMERICAN DENTAL SOCIETY OF EUROPE.

### MEETING AT VEVEY, SWITZERLAND, AUG. 26, 27, AND 28, 1884.

REPORTED FOR THE INDEPENDENT PRACTITIONER BY DR. W. ST. GEO. ELLIOTT, OF LONDON.

The twelfth annual meeting of this society was one of great pleasure and profit to its members, and has left behind it memories that will not soon fade away. Dr. W. D. Miller, of Berlin, the President of the society, occupied the chair, and Dr. F. Foerster, of the same city, was Secretary. After the fraternal greetings of compatriots in a foreign land were over, and the necessary preliminary business disposed of, the secretary proceeded to read a paper by Dr. C. M. Wright, formerly of Basel, Switzerland, now of Cincinnati, Ohio, entitled "Clinical Observations and Laboratory Experiments." (See page 613.)

*Dr. Du Bouchet*, of Paris—The paper is very flattering to us as a society, but I fear ranks us too highly.

*Pres. Miller*—Perhaps that is true, but it should be remembered that Dr. Wright was always very enthusiastic over a society of which he was one of the founders.

*Dr. Elliott*, of London—As some of the subjects mentioned in the paper will of necessity come up in the consideration of other papers that are to be read, I suggest that the discussion be postponed until all can be debated together.

*Pres. Dr. Miller*—It is our pleasure, gentlemen, to have a well known investigator from the other side with us to-day, and as he has some decided opinions regarding Riggs' disease, so-called, I will ask him to give us his views.

*Dr. Bodecker*, of New York—I am glad to meet with the American Dental Society of Europe, but I do not know that I have anything to say that is new or of special value. My views have been fully detailed in the reports of the Odontological Society of New York, but they have not always been correctly reported. I will give a short review of my opinions and experiences. The essential feature of Riggs' disease, or more properly Pyorrhœa Alveolaris, consists of pus flowing from the sockets about the teeth, such pockets often being the result of tartar, or arising from a lack of thorough cleansing on the part of the patient. The disease is found at all ages, but more frequently late in life; I have, however, seen it in a child of eight years of age, the teeth being elongated—one or two nearly one-fourth of an inch. The most common cause, particularly late in life, is tartar. Systemic causes are by no means absent, certain temperaments being more liable than others. Hereditary transmission may also be a factor of some moment in the case. As time will not permit me to consider the question of sources fully, I will confine my remarks to the disease as we ordinarily see it when induced by tartar or a lack of care or cleanliness.

1st. Remove all the pieces of tartar that are fairly accessible without too seriously wounding the gum. Some good operators, like Dr. Abbott, claim that they meet with the most success when strictly following the lines marked out by Riggs, but I think this an improper apprehension of the conditions found in this disease. If an operator were to remove all the tartar in the first stage of the disease, where there are loose gums, pockets and pus, it may be all that is necessary, but the probabilities are that these pockets will again become filled with *débris*, etc., and the disease make further progress. What should then be done? I would, as stated before, gently remove all foreign matter, wash out the pockets with a solution of corrosive sublimate, 1 part to 1000 of water, and if I suspected necrotic tissue under the gums I would use a saturated solution of sulphate of zinc, or a saturated solution of iodide of potash, with crystals of iodine added, applying the remedies with a stiff nerve plugger wound with cotton, carrying it up under the gums. Necrotic tissue shows itself by a dark blue line along the margins of the gums. On the following day, if the color is still present, renew the application; it generally disappears after two or three applications. Then I would apply the bi-tartrate of chinoline, which is an antiseptic and astringent, but not an irritant; one part to forty-five of water I find to be about the right strength; repeat the application until the gums look healthy. After an interval of several days again apply as before. The cure is generally complete in three or four weeks. I have had some very bad cases, the teeth so loose that they could be readily removed by the finger. Extreme cases of this kind require great care, not only on the part of the dentist but also on that of the patient, for the teeth must be thoroughly cleaned after every meal. To assist in this I prescribe some antiseptic wash. At each visit I remove more and more of the tartar, then support the loose teeth either by a silk ligature, or what is better, a fine gold wire. I think it was Dr. Clark, of New York, who recommended the use of a support of oxy-phosphate of zinc; this may be done, or a small plate made after taking an impression in plaster, the loose teeth having been tied together previously. It is important that this plate should not rest upon nor impinge upon the gums, as perfect cleanliness could not then exist. I give the patient a syringe and a solution of peroxide of hydrogen, directing that the pockets about the gums be well washed with the medicament. Of course we must not expect success in every case, but I find that where the treatment indicated is carried out, seventy per cent. of the cases will be cured.

*Dr. Du Bouchet*—I have sometimes used a small supporting plate in the cases spoken of by Dr. Bodecker, and with benefit; the plate resting on the gums.

*Dr. Bodecker*—Then you cannot have cleanliness, for tartar will gather under the plate, and thus irritate the gums.

*Dr. Du Bouchet*—The object is partly to get the tartar deposited on the plate, and thus save the gums.

*Dr. Bodecker*—It is much better not to have the plate touch the gums at all; the patient should come once in a while to have the plate removed and thoroughly cleaned.

*Dr. De Trey*—I have used these supports for loose teeth for about ten years. After thoroughly removing tartar I take a long piece of fine gold wire, made of pure gold, and commencing at one of the back teeth I weave it backward and forward, in and out where there is room, twisting it where it passes between the teeth around to the other side, thus firmly supporting all the teeth embraced, and yet not interfering with cleanliness.

*Dr. Bodecker*—That is about the way I do it.

*Dr. Terry*, of Zurich—When you find the teeth elongated can you reduce them?

*Dr. Bodecker*—Yes, if I can get any antagonism; otherwise I have no hope of their remaining in their proper position.

*Dr. Sachs*, of Breslau—That is my experience.

*Dr. Gregory*, of Lyons—Do you remove all superfluous liquid when you are treating the gums?

*Dr. Bodecker*—Yes, with bibulous paper.

*Dr. Williams*, of Geneva—Is dryness necessary?

*Dr. Bodecker*—Certainly.

*Dr. Sachs*—When the disease is somewhat dependent upon malarticulation I have found it hopeless to medicate.

*Dr. Bodecker*—I do not give up any case when the pulp is alive.

*Dr. Sachs*—How do you know when the pulp is dead?

*Dr. Bodecker*—By the color, stomatoscope, electric light, etc. You may allow a molar to remain even if one root is detached by the disease.

*Dr. Sachs*—The Countess of —— had formerly been under Dr. Miller's care; her teeth were elongated, with inflamed gums, bad breath, etc. The case was relieved, but the disease recurred.

*Dr. Miller*—The case reported by Dr. Sachs is a very peculiar one. The patient had never been troubled in any way with her teeth till one day when she bit in two a piece of green yarn. She at once experienced a very disagreeable taste, followed in a very few hours by severe pain of the incisor teeth above and below, and an inflammation of the gums which confined her to bed for nearly two weeks. As the inflammation subsided, suppurating pockets were formed on the right central incisors above and below. Whether the disease was caused by some poison in the yarn I am unable to say.

*Dr. Chamberlain*, of Rome—I would like to ask Dr. De Trey how long he would use gold ligatures on loose teeth.

*Dr. De Trey*—If the case is bad I keep the string on always. My father-in-law is an example. I fastened his teeth that way three times. Once one tooth came off; I cut a groove around it and tied it in with the others, and he has kept it for ten years, thus doing without artificial teeth.

*Dr. Du Bouchet*—This subject is a most interesting one; we must learn from each other. I have used instruments and adopted the radical treatment, but only with partial success. I syringe out the pocket with oxygenated water, using a probe-pointed syringe to push away the gums in advance of the liquid. I then apply iodide of zinc in limited quantity, as I found in one case extensive inflammation resulting from its too liberal employment; then I use a weak solution of carbolic acid (five per cent), direct the patient to use a soft brush, and to return every day. We must persevere in such cases; the disease will return if we do not persist. I have had some severe cases; in one, the lower canine and incisor teeth were badly affected. I could not get at the pockets, they were so deep. You may remember that at the meeting of the society held two years ago at Ostend, I exhibited an electric cautery; this apparatus I have found very useful. In this case I cauterize the gum from the free margin down to the pocket. The wound healed with astonishing rapidity. I desire to ask Dr. Bodecker if he has had any experience with this instrument.

*Dr. Bodecker*—No. I omitted to mention that in some cases there is partial necrosis of alveolus. Do what you will in the ordinary way and you meet with no success; it is then necessary to use a Riggs scaler, going deeply down and removing the offending part. Only in these isolated cases have I found heroic treatment necessary.

*Dr. Edwards*—I have used chloride of zinc in this disease, alternating with aromatic sulphuric acid; but in the ligation of loose teeth my experience has been unfortunate, as I find that I am likely to pull the teeth out. Acute cases are easily treated; it is not so with the chronic. In acute forms of the disease I use boracic acid and a weak solution of chloride of zinc. I would like to ask Dr. Du Bouchet whether he still continues to think well of sulphate of copper.

*Dr. Du Bouchet*—I do not use it as much as formerly.

*Dr. Edwards*—I prefer wood, with a bit of cotton wrapped around it, as a vehicle to make the application to the gums.

*Dr. Bodecker*—I have also found boro-glycerole useful, but do not advise too large a pharmacopœia. If we give the gums the necessary conditions they will recover. A wound on the hand will not heal if it is flooded with saliva and other secretions; so when you give nature a chance with the gums she will do her duty.

*Dr. Edwards*—I have found the tincture of sumach a good astringent in the after treatment.

The meeting then adjourned.

(TO BE CONTINUED.)

